# IDH status shapes glioma oncotopy: voxel-wise mapping of 644 adult diffuse gliomas

**DOI:** 10.1007/s00234-026-03991-0

**Published:** 2026-05-07

**Authors:** Freya Garhöfer, Yeong Chul Yun, Sabine Wolf, Katharina Holz, Johann M. E. Jende, Anja Hohmann, Philipp Vollmuth, Martin Bendszus, Corrado Santarosa, Karl-Olof Lövblad, Heinz-Peter Schlemmer, Felix Sahm, Sabine Heiland, Varun Venkataramani, Felix T. Kurz

**Affiliations:** 1 Faculty of Medicine, Heidelberg University, Heidelberg, Germany; 2 Department of Neuroradiology, University Hospital Heidelberg, Heidelberg, Germany; 3 Division of Radiology, German Cancer Research Center (DKFZ), Heidelberg, Germany; 4 Department of Neurology, University Hospital Heidelberg, Heidelberg, Germany; 5 Department of Neuroradiology, University Hospital Bonn, Bonn, Germany; 6 Department of Neuroradiology, University Hospital of Geneva, Geneva, Switzerland; 7 Department of Neuropathology, University Hospital Heidelberg, Heidelberg, Germany; 8 Clinical Cooperation Unit Neuropathology, German Cancer Consortium (DKTK) German Cancer Research Center (DKFZ), Heidelberg, Germany; 9 Department of Functional Neuroanatomy, Heidelberg University, Heidelberg, Germany

**Keywords:** Glioblastoma, Glioma, MRI, Astrocytoma, Oligodendroglioma, Pre-treatment diagnosis

## Abstract

**Background:**

The aim of this study was to characterize the anatomical and imaging features of adult-type diffuse gliomas across histomolecular subtypes.

**Methods:**

Clinical 3-Tesla brain MRI images from 644 patients with pathologically confirmed adult-diffuse glioma before treatment was retrospectively evaluated: 527 IDH-wildtype glioblastoma, 71 astrocytoma, and 46 oligodendroglioma. Pre- and post-contrast T1-weighted, T2-weighted and FLAIR sequences were part of the MRI protocol. Contrast-enhancing tumors and non-enhancing lesions (NEL) were automatically segmented using HD-GLIO. We used a voxel-wise Fisher-exact-test followed by random-permutation (ADIFFI) to identify regions with higher occurrence of tumor associated with IDH-mutation status or 1p/19q-codeletion status. Mann-Whitney-U-test was used to compare signal intensities in CET and NEL across the three different subtypes of adult-diffuse glioma investigated here.

**Results:**

We observed a significant correlation of IDH-mutant gliomas with a predominance in the frontal lobe adjacent to the rostral extension of the lateral ventricles. IDH-wildtype tumors had larger NEL volumes than IDH-mutant gliomas (*p* < 0.0001). Signal intensity analysis demonstrated consistently lower T1w and T1-CE values and higher T2w and FLAIR values in IDH-mutant gliomas (all *p* < 0.0001). Oligodendrogliomas showed higher signal intensity on T1-CE images compared to astrocytomas (*p* = 0.035).

**Conclusion:**

We analyzed a large radio-genomic patient cohort consisting of glioblastoma, astrocytoma and oligodendroglioma. Our findings are in line with previously analyzed smaller patient cohorts. Our findings underline the importance of the IDH-mutation for determining tumor location and potentially point to a cell of origin along the rostral extension of the lateral ventricles.

**Supplementary Information:**

The online version contains supplementary material available at 10.1007/s00234-026-03991-0.

## Key points

Adult-type diffuse gliomas with IDH-mutation showed a predominance in the frontal lobe adjacent to the rostral extension of the lateral ventricles.

Signal intensity analysis demonstrated consistently lower T1w and T1-CE values and higher T2w and FLAIR values in IDH-mutant gliomas.

Oligodendrogliomas showed higher mean signal intensity of NEL on T1-CE images than astrocytomas.

**Supplementary Information:**

The online version contains supplementary material available at 10.1007/s00234-026-03991-0.

## Introduction

Diffuse gliomas are among the most common primary brain tumors in adults and display a wide spectrum of clinical and radiological characteristics. The 2021 WHO classification emphasizes the prognostic relevance of molecular markers, particularly isocitrate-dehydrogenase (IDH) mutation status and 1p/19q-codeletion, to distinguish glioblastoma (IDH-wildtype), astrocytoma (IDH-mutant), and oligodendroglioma (IDH-mutant, 1p/19q-codeleted) as distinct disease entities [1]. These molecular subtypes not only vary in prognosis and treatment response, but also exhibit differences in growth dynamics, anatomical localization, and imaging characteristics [2–4].

Previous studies have shown that IDH-wildtype gliomas are more likely to exhibit diffuse infiltration, peritumoral hyperintensities in T2-weighted and fluid-attenuated inversion recovery (FLAIR) images, and preferential localization in functionally eloquent regions, including perisylvian and subcortical structures [5]. In contrast, IDH-mutant gliomas often present with more circumscribed lesions and tend to occur in the frontal lobes or lateral temporal regions [2]. These topographical patterns are thought to reflect underlying biological differences in tumor pathogenesis [6, 7].

Despite increasing emphasis on imaging biomarkers in glioma classification and treatment monitoring [8–10], the spatial relationship between molecular subtype, lesion localization, and clinical outcome remains incompletely understood. In particular, non-enhancing lesion (NEL) components visible on T2w/FLAIR imaging may harbor infiltrative tumor beyond the contrast-enhancing core and have been associated with both recurrence risk and functional decline [9, 11, 12].

Advanced voxel-wise statistical mapping techniques, such as the Analysis of Differential Involvement (ADIFFI), originally introduced by Ellingson et al. [13, 14], enable systematic evaluation of spatial tumor distribution across molecular subtypes in large cohorts.

Additionally, quantitative analysis of MRI signal intensities and lesion volumes may help distinguish tumor biology in a non-invasive manner [10, 15]. The present study aims to characterize the anatomical and imaging features of adult-type diffuse gliomas across histomolecular subtypes according to the most recent definition of the 2021 WHO classification of tumors of the central nervous system. Using a large retrospective cohort, standardized intensity scaling, and permutation-controlled voxel-wise ADIFFI mapping, we examined differences in tumor localization, lesion volume, and MRI signal profiles between IDH-wildtype glioblastomas, astrocytomas, and oligodendrogliomas.

## Materials and methods

### Patients

Retrospective evaluation of imaging data was approved by the local ethics (local identifiers: S-320/2012 and S-784/2018). Informed consent was waived due to the retrospective nature of the study and the thorough anonymization of the data. Patients with diffuse glioma were recruited between April 2010 and March 2022 from a single center of the Department of Neuroradiology of a university hospital in Germany. A total of 796 patients were identified with a newly diagnosed diffuse glioma. In this retrospective study, patients were included if the following two criteria were met: (1) glioma was confirmed by pathology and molecular parameters including IDH mutation status and 1p/19q-codeletion status were reported, (2) pre- and post-contrast T1-weighted (T1w and T1-CE respectively), T2-weighted (T2w) and FLAIR images were available before treatment had been started. 50 patients were excluded from the analysis due to incomplete information regarding IDH-mutation status and/or 1p/19q-codeletion status. Furthermore, 102 patients were excluded from our retrospective analysis because of insufficient image quality in at least one of the radiological images. Therefore, 644 patients with newly diagnosed diffuse glioma were included in our retrospective evaluation.

## Magnetic resonance imaging

Every patient received standardized 3 Tesla MRI examination of the brain using a 12-channel head-matrix coil from one of these devices: Magnetom Trio TIM, Prisma fit, Verio or Skyra (all from Siemens Healthineers AG, Germany). As described previously [12, 16], the following four MRI sequences were included in the protocol: T1-weighted images before (T1w) and after (T1-CE) a gadolinium-based contrast agent administration, a T2-weighted image (T2w) and an image with fluid attenuated inversion recovery (FLAIR). A 3D magnetization-prepared rapid acquisition with gradient echo (MPRAGE) sequence was used to obtain T1w and T1-CE. Gadoterate meglumine (Dotarem^®^, Guerbet, France) with a dose of 0.1 mmol/kg was administered as a MR contrast agent for T1-CE. For both axial T2w and axial FLAIR the parameters the section thickness and the spacing were chosen as 5 mm and 5.5 mm respectively. Important measurement parameters for each MRI sequence are included in Table 1.

**Table 1:** MRI measurement parameters at 3 Tesla for obtaining pre-contrast T1-weighted (T1w), T2-weighted (T2w), post-contrast T1-weighted (T1-CE), and fluid-attenuated inversion recovery (FLAIR) MRIs are shown

MR parameters	T1w/T1-CE	T2w	FLAIR
Inversion time (TI) in ms	900–1100	-	2400–2500
Echo time (TE) in ms	3–4	85–88	85–135
Repetition time (TR) in ms	1710–2250	2740–5950	8500–10,000
Flip angle in degrees	15	170–180	170–180
Section thickness in mm	1	5	5
Spacing in mm	0	5.5	5.5

## Image post-processing

Brain extraction and segmentation of glioma were performed automatically using HD-BET [17] and HD-GLIO [18, 19]. Masks of contrast-enhancing tumor (CET) regions in T1-CE and non-contrast-enhancing lesion (NEL) in FLAIR were obtained after segmentation of tumor lesions. Visual inspection of segmented masks was performed by two board-certified neuro-radiologists to assess the accuracy of automatic segmentation. The segmentation masks were manually corrected in consensus if the segmentation was inaccurate which was necessary for 28 cases (3.5% of total cases). Furthermore, signal intensity values in all MRI images were normalized by using white-stripe-normalization [20]. Brain-extracted T1w from all patients were registered to a high-resolution (1.0 mm isotropic) T1w brain atlas from the Montreal Neurological Institute (MNI) (template: MNI-152-9c [21]) using the linear image registration tool of FSL with 12 degrees of freedom for transformation [22, 23]. The transformation matrix resulted from this step was applied to transform the tumor masks to the common MNI space.

## Frequency maps and analysis of differential involvement (ADIFFI)

Lesion frequency maps were obtained by grouping the patients by their tumor mutation status. Here, frequency maps of ROIs were computed by superimposing every mask of that ROI within a group in a common MNI-152 space and normalizing it by the number of patients in that group. The frequency maps of both groups were subtracted to obtain a map of difference in lesion frequencies. Fisher’s exact-test was applied voxel-wise to identify brain regions where the occurrence of a tumor lesion significantly differed between two groups. Here, each voxel in the MNI brain is first assigned “lesion present” or “not present” for each subject, creating frequency maps per group. Then, ADIFFI performed a Fisher’s exact test separately at each voxel to see where the occurrence differs significantly between groups. Cluster-based permutation correction [24] was used here as multiple comparison correction to control the false discovery rate. Here, for each permutation, segmented masks were assigned randomly to one of two groups mentioned above while keeping the marginal population in each group constant. Voxel-wise Fisher’s exact-test was performed after this random assignment and voxel p-values that were less than 0.05 were retained in the ADIFFI results (with α = 0.05, power > 0.8). FSL cluster [23] was used to identify clusters with voxel-connectivity of 26 as proposed by others [12, 13, 25, 26]. The maximum cluster size was recorded after each permutation. As described previously [12, 13, 25, 26], 500 permutations were performed to estimate the cluster-size threshold as the 95-percentile of the maximum cluster size after 500 permutations.

### Statistical analysis

The non-parametric Mann-Whitney U test was used for comparing age, lesion volume and mean signal intensities of tumor lesions. For the mean signal intensity of the tumor lesion, the mean value of signal intensity value from every voxel within the NEL were computed. Outliers in volume data were identified and removed with the ROUT method (Q = 1%) in GraphPad Prism v10.0.2 (GraphPad Software Inc., Boston, USA). A p-value of less than 0.05 was reported as a significant difference.

## Results

### Patient cohort

**Fig. 1:**
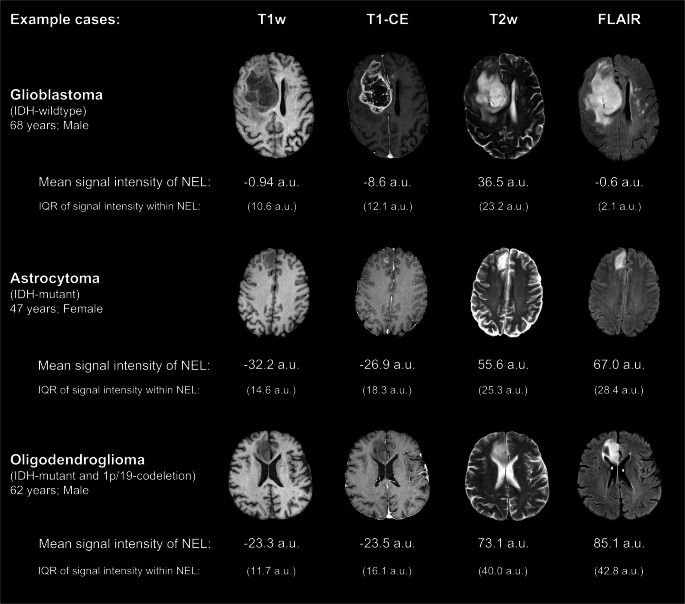
Example cases for each entity of adult-type diffuse glioma from our study cohort are presented here. Furthermore, the mean and interquartile range (IQR) of signal intensity values from non-contrast enhancing lesions (NEL) are shown here after each image is normalized by using white-stripe normalization in arbitrary unit (a.u.)

A total of 644 patients with adult-type diffuse gliomas were included in this retrospective study. Among them, 527 patients were diagnosed with IDH-wildtype glioblastoma (WHO grade 4), and 117 with IDH-mutant lower-grade gliomas, comprising 71 astrocytomas (WHO grade 3: *n* = 23, WHO grade 2: *n* = 48) and 46 oligodendrogliomas (WHO grade 3: *n* = 8, WHO grade 2: *n* = 38) defined by concurrent 1p/19q-codeletion. Patients with (IDH-wildtype) glioblastoma were older than those with IDH-mutant gliomas (62.6 ± 12.0 vs. 40.1 ± 12.9 years, *p* < 0.0001). Example cases for each entity of adult-type diffuse glioma are presented in Fig. 1.

## Tumor localization pattern

**Fig. 2:**
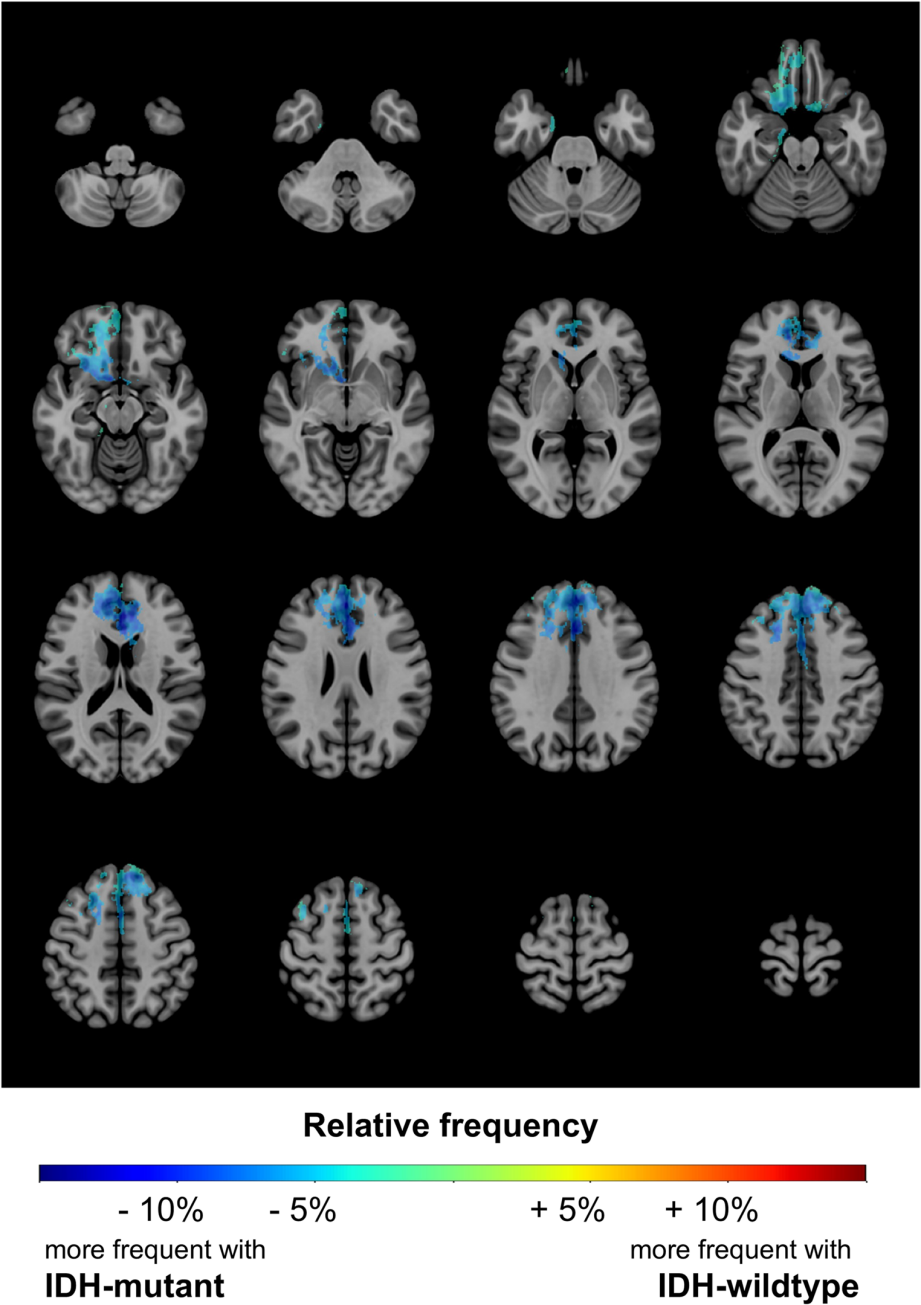
ADIFFI-based cluster map showing significant differences in tumor localization between IDH-wildtype and IDH-mutant gliomas. Only clusters are shown here, which were significant after ADIFFI analysis. ADIFFI analysis resulted in one significant cluster (cluster size: 90722 voxels, *p* < 0.001)

Voxel-wise analyses revealed distinct spatial distributions across molecular subtypes. IDH-wildtype GBMs occurred more frequently in perisylvian, insular, and deep subcortical structures like in basal ganglia and internal capsule. In contrast, IDH-mutant gliomas showed a predilection for frontal pole and lateral temporal regions adjacent to the subventricular zone (SVZ). These findings were confirmed by the ADIFFI framework with cluster-level permutation correction, indicating statistically significant localization differences between groups (Fig. 2). No ADIFFI-significant clusters were found between astrocytomas and oligodendrogliomas.

### Tumor volume and MRI signal characteristics

**Fig. 3:**
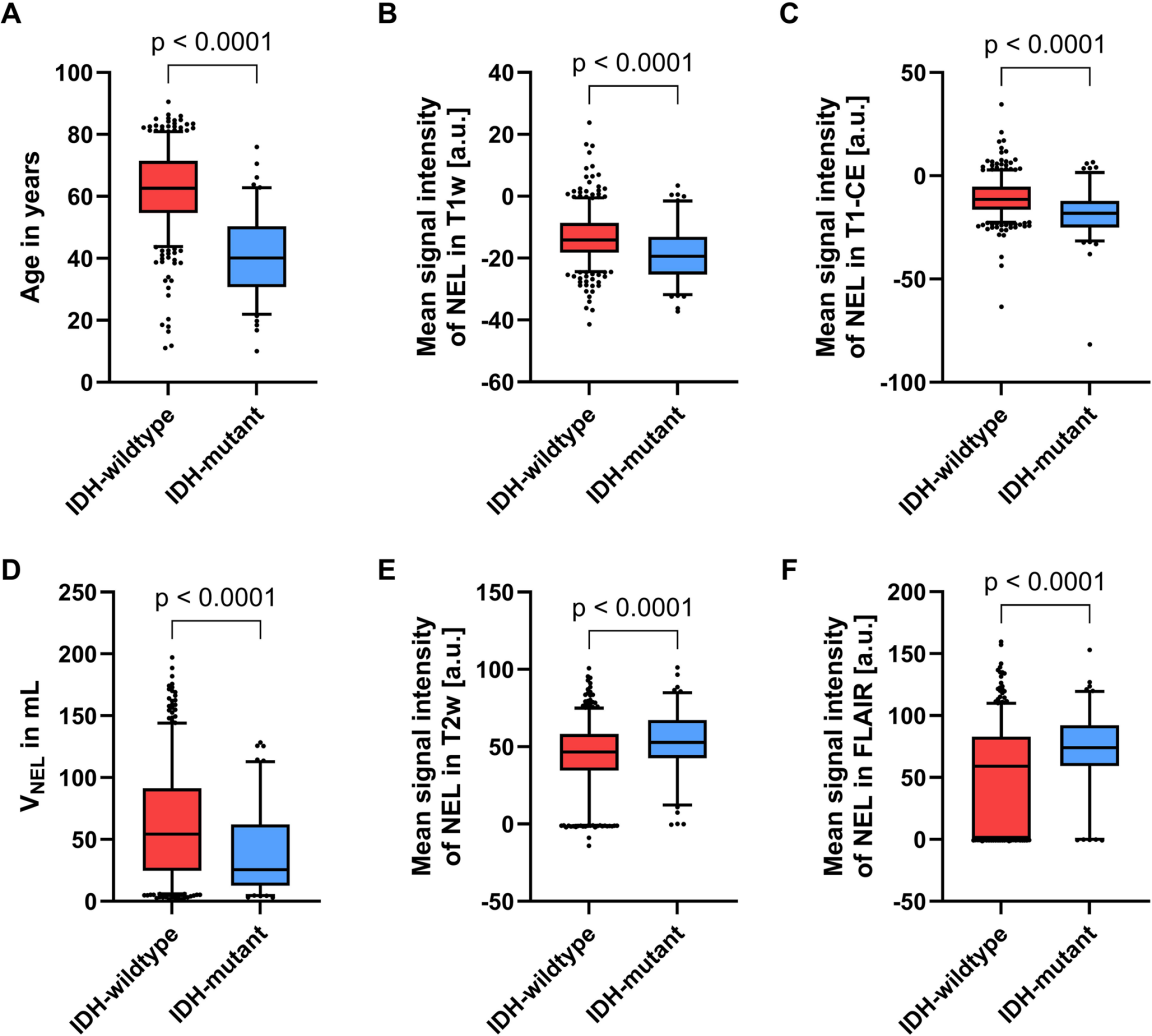
Boxplots comparing (**A**) age at diagnosis, (**D**) non-enhancing lesion (NEL) volume (V_NEL_), and normalized MRI signal intensities of NEL in (**C**) pre-contrast T1-weighted (T1w), (**C**) contrast-enhanced T1w (T1-CE), (**E**) T2-weighted (T2w), and (**F**) FLAIR images between IDH-wildtype and IDH-mutant gliomas

**Fig. 4:**
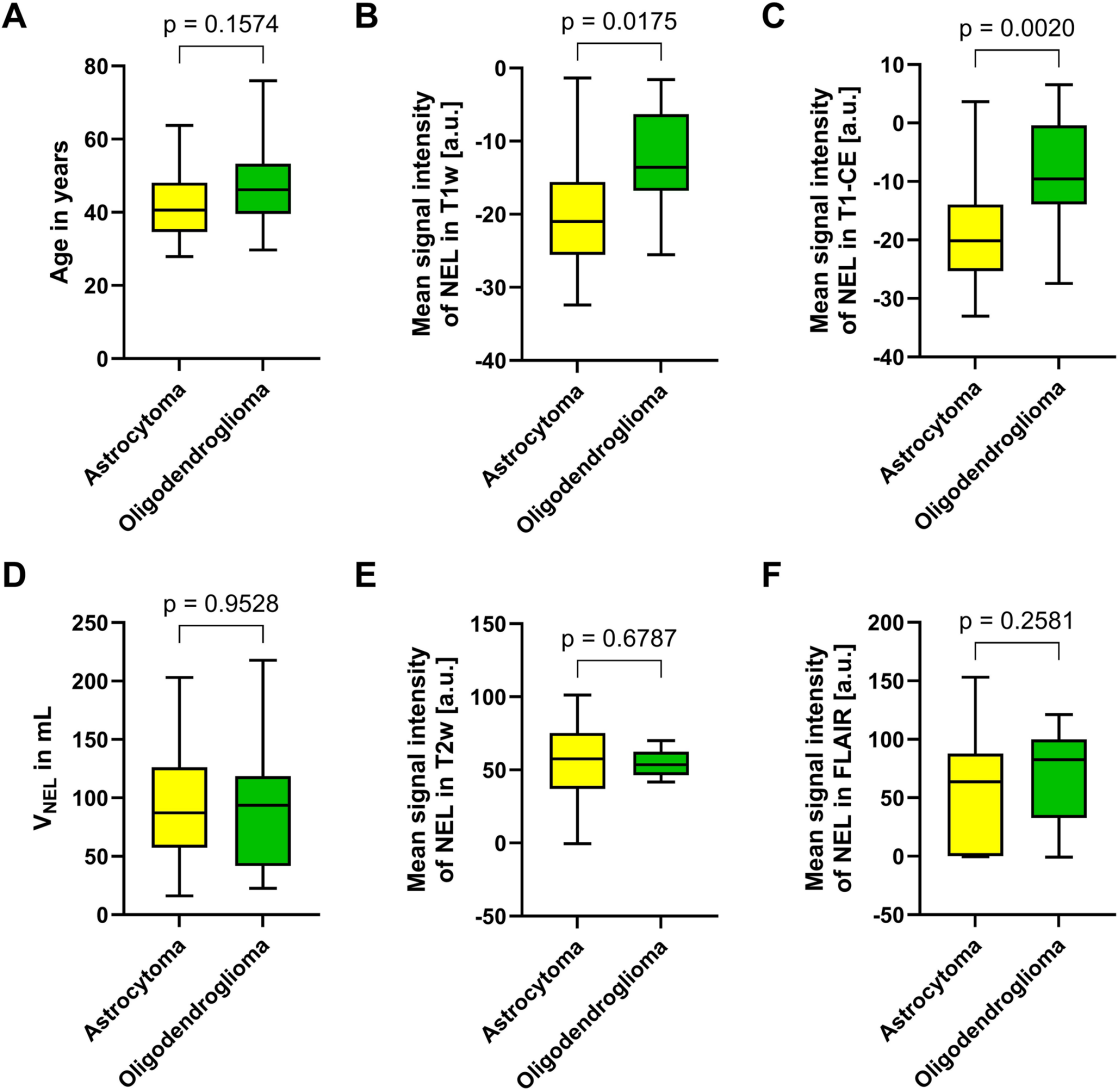
Boxplots comparing (**A**) age at diagnosis, (**D**) non-enhancing lesion (NEL) volume (V_NEL_), and normalized MRI signal intensities of NEL in (**C**) pre-contrast T1-weighted (T1w), (**C**) contrast-enhanced T1w (T1-CE), (**E**) T2-weighted (T2w), and (**F**) FLAIR images between astrocytoma (IDH-mutant, without 1p/19q-codeletion) and oligodendroglioma (IDH-mutant, with 1p/19q-codeletion)

Quantitative comparisons of non-enhancing lesion (NEL) volumes and normalized MRI signal intensities revealed marked differences between IDH subtypes. IDH-wildtype tumors had larger NEL volumes (54.2 ± 44.0 ml) than IDH-mutant tumors (25.5 ± 35.2 ml, *p* < 0.0001). Signal intensity analysis demonstrated consistently lower T1w and T1-CE values and higher T2w and FLAIR values in IDH-mutant gliomas (all *p* < 0.0001) (Fig. 3). Out of 117 patients with IDH-mutant glioma 31 patients had a CET component present in MRI. Results comparing parameters extracted from CET were presented in Supplementary Figures S1 and S2. Within the IDH-mutant subgroup, no volume differences were observed between astrocytomas and oligodendrogliomas (42.6 ± 55.0 ml vs. 23.4 ± 49.8 ml, *p* = 0.68). However, oligodendrogliomas showed higher signal intensity on T1-CE images (−15.0 vs. −20.5, *p* = 0.035), while no differences were found for other sequences (Fig. 4). Additional analyses were performed for IDH-mutant glioma patient cohort stratified by tumor grade. Results were included in the supplementary materials: Tables S1 for grade 2, and Tables S2 for grade 3 IDH-mutant gliomas.

## Discussion

In this large retrospective cohort, conventional MRI phenotypes and spatial patterns consistently differentiated IDH-wildtype glioblastoma from IDH-mutant gliomas, while failing to robustly separate IDH-mutant astrocytoma from oligodendroglioma. Glioblastoma presented at older age, exhibited larger non-enhancing lesion (NEL) volumes, and showed a distinct signal-intensity constellation across T1w/T1-CE vs. T2w/FLAIR. Voxel-wise mapping revealed a predilection in the frontal lobe adjacent to the subventricular zone (SVZ) for IDH-mutant gliomas, whereas IDH-wildtype gliomas lacked a dominant locus. Together, these findings support a pragmatic diagnostic stance: routine MRI can raise or lower pretest probability for IDH status, but definitive subtyping within IDH-mutant disease requires molecular testing, particularly assessment of 1p/19q-codeletion [16, 27–29].

Our spatial findings align with prior reports that IDH-mutant gliomas preferentially involve the frontal lobe, particularly near the rostral horns of the lateral ventricles [16, 27]. In contrast, no consistent subtype-specific predilection distinguishing IDH-mutant astrocytomas from oligodendrogliomas has been demonstrated, as both share this SVZ-adjacent localization [30]. The absence of significant ADIFFI-clusters observed in our study between astrocytomas and oligodenrogliomas may be interpreted because of a smaller cohort size for IDH-mutant glioma. However, our observation is in line with the underlying biology as multiple other studies suggest that both types of IDH-mutant gliomas may share common progenitor origin resulting in similar brain region as predilection areas for IDH-mutant glioma [31, 32]. Age and volume contrasts also mirror earlier observations: IDH-wildtype gliomas occur at higher age and with larger non-enhancing compartments, whereas IDH-mutant gliomas are more frequent in younger adults as reported by others [29].

The signal-intensity constellation observed in our study is concordant with known pathophysiology: stronger angiogenesis/microvascular proliferation and a higher prevalence of hemorrhage and necrosis in glioblastoma [33, 34], and the T2w/FLAIR-mismatch phenomenon in subsets of IDH-mutant gliomas [33, 34]. Beyond replication, our study adds granularity by quantifying the larger NEL burden in IDH-wildtype tumors an imaging correlate of infiltrative biology with prognostic relevance [28]. Furthermore, by showing only modest differences between astrocytoma and oligodendroglioma, underscoring the limits of structural MRI alone for preoperative subtyping regarding 1p/19q-codeletion status [28]. The higher mean signal intensity values of the NEL from contrast-enhanced T1-weighted MRI observed in our study for oligodendrogliomas compared to astrocytomas seems to reflect the higher microvascular density observed by others for grade II oligodendrogliomas compared to grade II astrocytoma [35].

The shared SVZ-adjacent predilection of IDH-mutant gliomas is consistent with models positing neural stem/progenitor cells in the SVZ as a cell-of-origin niche [27, 36, 37]. By contrast, the diffuse behavior of IDH-wildtype glioblastoma fits a paradigm of widespread microscopic infiltration and possibly SVZ-seeded migratory clones [16, 38]. The observation that astrocytoma and oligodendroglioma shows only subtle spatial divergence supports a timing model: an early, spatially directing IDH event followed by later genetic branching (TP53/ATRX vs. 1p/19q-codeletion) that alters biology and prognosis without markedly relocating the initial footprint [16]. The frequent periventricular involvement of IDH-mutant gliomas and the biological role of neural stem cells in the SVZ support ongoing exploration of SVZ-informed radiotherapy concepts [39].

Several limitations need to be acknowledged for our retrospective study. First, our data were acquired at a single center on Siemens platforms; scanner/vendor/sequence heterogeneity is known to affect quantitative measurements especially regarding signal intensity values, arguing for multicenter harmonization and external validation. Second, we included only anatomical MRI sequences. Multimodal integration: combining structural MRI with diffusion, perfusion, and spectroscopy offers significant potential to improve molecular prediction (e.g., IDH status) [29, 40]. While recent studies demonstrate the benefits of combining these modalities, future work should aim to implement standardized pipelines and harmonized acquisition protocols to ensure generalizability across centers. Third, the diffuse, network-like spread characteristic of diffuse glioma challenges purely voxel-centric inference. Future work may integrate tractography and network-level models to capture system-scale tumor-brain interactions [41]. Lastly, the retrospective study cohort of IDH-mutant glioma patients mostly consisted of WHO grade 2 (74%) glioma and there was no astrocytoma WHO grade 4 in the study population. Therefore, it cannot be completely ruled out that our findings reflect tumor grade rather than IDH-mutation status. For a more useful clinical application in preoperative imaging evaluation, determining a threshold value of radiological parameters for distinguishing IDH-wildtype glioma and IDH-mutant gliomas might be desirable. However, given the biological continuum of imaging characteristics and overlap between IDH-wildtype and IDH-mutant gliomas, we deliberately avoided dichotomization to prevent loss of information and potential reduction in predictive performance. Identifying ideal cut-off values for this problem can be addressed in a future study with a larger patient cohort and in a multi-centric approach for external validation of ROC curve analysis.

## Conclusion

In a large cohort, conventional MRI combined with voxel-wise mapping robustly separates IDH-wildtype from IDH-mutant gliomas by age, NEL burden, signal phenotype, and spatial predilection, but does not reliably discriminate astrocytoma from oligodendroglioma. These findings support a workflow in which routine MRI guides the pretest probability of IDH status, while definitive subtyping relies on molecular diagnostics. The consistent frontopolar/subventricular-adjacent pattern of IDH-mutant disease and the diffuse, system-level spread of IDH-wildtype glioblastoma reflect distinct biological programs with diagnostic and therapeutic implications that future multimodal, multicenter, and prospective studies should harness.

## Supplementary Information

Below is the link to the electronic supplementary material.

Supplementary Material 1

## Data Availability

The MRI scans used in this study, obtained from Heidelberg University Hospital, are not publicly available due to institutional and ethical regulations.

## Abbreviations

ADIFFI: Analysis of Differential Involvement
a.u.: arbitrary unit
CET: contrast-enhancing tumor
FLAIR: fluid attenuated inversion recovery
IDH: isocitrate-dehydrogenase
NEL: non-enhancing lesion
MNI: Montreal Neurological Institute
SVZ: subventricular zone
T1w: T1-weighted
T1-CE: T1-weighted contrast-enhanced
T2w: T2-weighted
