# Supplementary material for: IDH status shapes glioma oncotopy: voxel-wise mapping of 644 adult diffuse gliomas
Source: Neuroradiology. 2026 May 7;68(6):1717–27. doi: 10.1007/s00234-026-03991-0 (PMC13323361; doi:10.1007/s00234-026-03991-0)
Supplement: Supplementary file 1 — Supplementary Material 1 [file 234_2026_3991_MOESM1_ESM.pdf]

Supplementary Materials

IDH status shapes glioma oncotopy:

voxel-wise mapping of 644 adult diffuse gliomas

Figure S1: Boxplots comparing (**A**) contrast-enhancing lesion (CET) volume (V_{CET}), and normalized MRI signal intensities of CET in (**B**) pre-contrast T1-weighted (T1w), (**C**) contrast-enhanced T1w (T1-CE), (**D**) T2-weighted (T2w), and (**E**) FLAIR images between glioblastoma (IDH-wildtype) and IDH-mutant glioma.

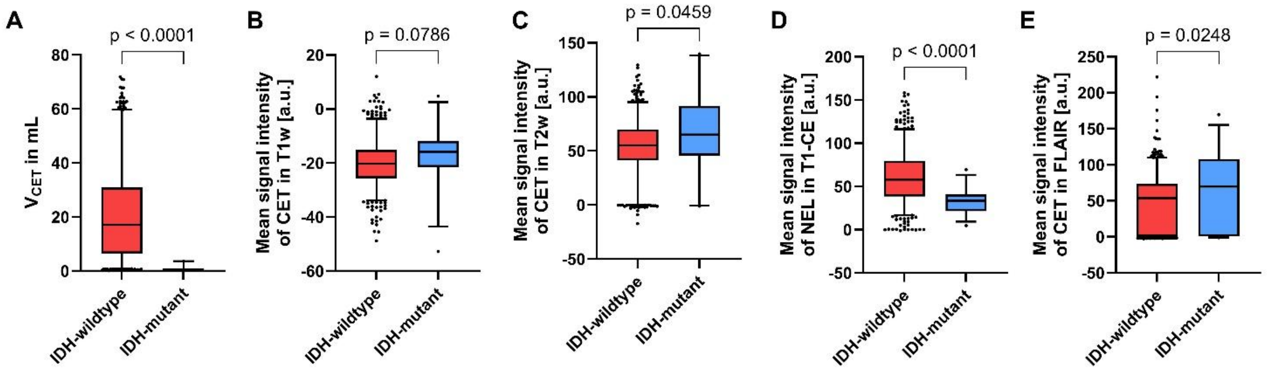

Figure S2: Boxplots comparing (**A**) contrast-enhancing lesion (CET) volume (V_{CET}), and normalized MRI signal intensities of CET in (**B**) pre-contrast T1-weighted (T1w), (**C**) contrast-enhanced T1w (T1-CE), (**D**) T2-weighted (T2w), and (**E**) FLAIR images between astrocytoma (IDH-mutant, without 1p/19q-codeletion) and oligodendroglioma (IDH-mutant, with 1p/19q-codeletion).

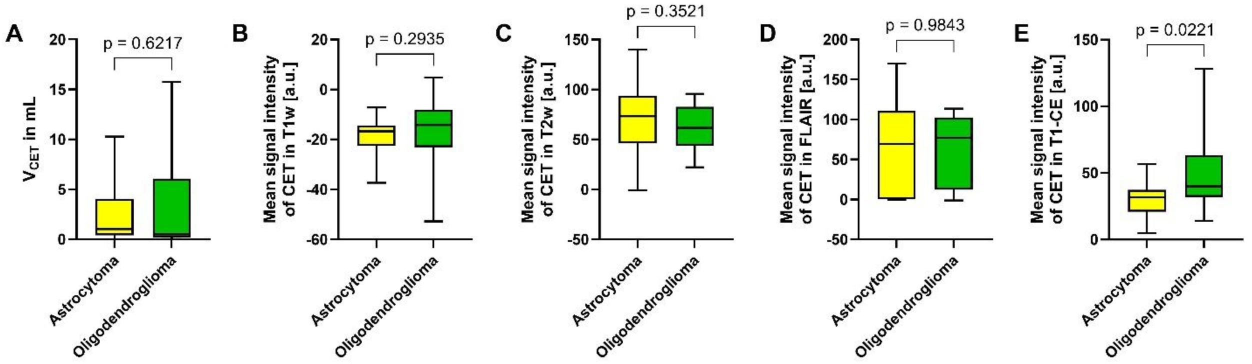

Table S1: Comparing age at diagnosis, non-enhancing lesion (NEL) volume (V_{NEL}), contrast-enhancing tumor (CET) volume (V_{CET}), and normalized MRI signal intensities of NEL and CET in pre-contrast T1-weighted (T1w), contrast-enhanced T1w (T1-CE), T2-weighted (T2w), and FLAIR images between astrocytoma (IDH-mutant, without 1p/19q-codeletion) and oligodendroglioma (IDH-mutant, with 1p/19q-codeletion) both WHO grade 2.

**WHO Grade 2**					
	Astrocytoma (IDH-mutant, no 1p/19q-codeletion)		Oligodendroglioma (IDH-mutant, 1p/19-codeleted)		p-value
	Mean	Standard deviation	Mean	Standard deviation	
Age [years]	40.38	13.07	42.59	14.42	0.58
V_{NEL} [ml]	52.49	58.61	39.50	45.95	0.61
Signal intensity in NEL in T1w [a.u.]	-20.45	8.253	-16.88	8.65	0.075
Signal intensity in NEL in T2w [a.u.]	56.49	20.38	51.78	15.87	0.34
Signal intensity in NEL in T1-CE [a.u.]	-21.03	12.27	-15.71	9.20	**0.049**
Signal intensity in NEL in FLAIR [a.u.]	68.54	35.71	75.12	27.66	0.36
V_{CET} [ml]	2.44	3.051	2.22	5.48	0.96
Signal intensity in CET in T1w [a.u.]	-18.53	6.50	-15.27	16.08	**0.038**
Signal intensity in CET in T2w [a.u.]	84.56	25.78	53.82	20.27	**0.015**
Signal intensity in CET in T1-CE [a.u.]	31.46	16.30	52.10	27.16	0.065
Signal intensity in CET in FLAIR [a.u.]	82.77	71.06	56.37	46.65	0.28

Table S2: Comparing age at diagnosis, non-enhancing lesion (NEL) volume (V_{NEL}), contrast-enhancing tumor (CET) volume (V_{CET}), and normalized MRI signal intensities of NEL and CET in pre-contrast T1-weighted (T1w), contrast-enhanced T1w (T1-CE), T2-weighted (T2w), and FLAIR images between astrocytoma (IDH-mutant, without 1p/19q-codeletion) and oligodendroglioma (IDH-mutant, with 1p/19q-codeletion) both WHO grade 3.

**WHO Grade 3**					
	Astrocytoma (IDH-mutant, no 1p/19q-codeletion)		Oligodendroglioma (IDH-mutant, 1p/19-codeleted)		p-value
	Mean	Standard deviation	Mean	Standard deviation	
Age [years]	39.42	11.73	41.16	8.30	0.71
V_{NEL} [ml]	62.49	46.95	89.72	49.10	0.16
Signal intensity in NEL in T1w [a.u.]	-18.62	9.94	-17.33	8.24	0.44
Signal intensity in NEL in T2w [a.u.]	49.25	26.04	53.39	10.41	0.81
Signal intensity in NEL in T1-CE [a.u.]	-17.65	9.88	-14.34	7.22	0.26
Signal intensity in NEL in FLAIR [a.u.]	61.14	36.96	68.27	38.28	0.46
V_{CET} [ml]	2.555	3.23	5.65	6.64	0.37
Signal intensity in CET in T1w [a.u.]	-19.70	9.44	-18.51	13.79	0.77
Signal intensity in CET in T2w [a.u.]	58.16	44.39	70.83	26.77	0.51
Signal intensity in CET in T1-CE [a.u.]	29.43	10.67	50.44	45.00	0.44
Signal intensity in CET in FLAIR [a.u.]	48.29	43.67	65.22	43.64	0.31
